# Nanotechnology-driven delivery of dexamethasone for arthritis: The role of liposomes

**DOI:** 10.17179/excli2026-9360

**Published:** 2026-06-01

**Authors:** Kajal Kumari, Anil Pareek, Swapnil Sharma, Sachin Sharma, Vipin Saini, Shadma Wahab, Devesh U. Kapoor

**Affiliations:** 1 Department of Pharmacy, Banasthali Vidyapith, Banasthali-304022, Rajasthan, India; 2 Department of Pharmaceutics, Anand Pharmacy College, Anand-388001, Gujarat, India; 3 Department of Pharmaceutical Chemistry, Parul Institute of Pharmacy & Research, Vadodara-391760, Gujarat, India; 4 MM College of Pharmacy, Maharishi Markandeshwar (Deemed to be University), Mullana, Ambala-133207, Haryana, India; 5 Department of Pharmacognosy, College of Pharmacy, King Khalid University, Abha-61421, Saudi Arabia; 6 Dr. Dayaram Patel Pharmacy College, Bardoli-394601, Gujarat, India; 7 Centre for Research Impact & Outcome, Chitkara College of Pharmacy, Chitkara University, Rajpura-140471, Punjab, India

**Keywords:** liposomes, dexamethasone, glucocorticoid, targeted delivery, arthritis treatment, anti-inflammatory

## Abstract

Arthritis is one of the most prevalent chronic musculoskeletal disorders worldwide, affecting more than 300 million individuals and representing a leading cause of pain, disability, and reduced quality of life. The global burden of osteoarthritis and rheumatoid arthritis continues to rise due to population aging, sedentary lifestyles, and increasing metabolic comorbidities. Liposomal drug carriers hold great promise in maximizing dexamethasone's therapeutic utility while minimizing its first-pass effect and systemic toxicity. Due to their biocompatibility, slow-release capability, and potential for target-specific delivery, liposomes enable localized drug sequestration within inflamed joints through both passive and active targeting mechanisms. This review aims to analyze the pharmacological action of dexamethasone in arthritis in conjunction with the advantages inherent to liposomal formulations, as well as recent advancements in liposome design, such as stimuli-responsive and theranostic liposomes. Despite their great promise, limitations, including drug leakage, immunogenicity, and regulatory hurdles, remain major impediments to their clinical use. Future directions indicate promise for personalized, image-directed liposomal therapies in a paradigm shift for arthritis treatment. Overall, liposomal dexamethasone represents a major breakthrough in the safe design of target-specific, effective anti-inflammatory therapies for arthritis.

See also the graphical abstract(Fig. 1).

## 1. Introduction

### 1.1 Overview of arthritis and its pathophysiology

Arthritis is a collection of more than 100 rheumatic diseases and disorders that primarily involve joints and result in disability in movement, inflammation, rigidity, and discomfort (Fitton and Melville, 2019[24]). The most prevalent are osteoarthritis (OA), rheumatoid arthritis (RA), and psoriatic arthritis. OA is characterized mostly as a degenerative joint disease characterized by cartilaginous degeneration (Mobasheri and Batt, 2016[49]). RA is an autoimmune disease caused by chronic inflammation involving the synovial membrane, which in itself destroys joints (Gao et al., 2024[25]). Both involve dominant roles for inflammation in disease pathology. Prominent mediators, including pro-inflammatory cytokines (e.g., TNF-α, IL-1β, IL-6), prostaglandins, and matrix metalloproteinases (MMPs), are responsible for joint destruction and extrinsic clinical manifestations. In RA infiltration, of T cells, B cells, and macrophages into synovial tissues, amplifies the inflammatory cascade, which in turn leads to synovial hyperplasia (Mueller et al., 2021[50]). This pathological remodeling leads to progressive cartilage erosion and bone destruction, ultimately resulting in severe disability if left untreated.

### 1.2 Limitations of conventional treatments

Conventional treatments for arthritis usually involve the use of non-steroidal anti-inflammatory drugs (NSAIDs), corticosteroids, disease-modifying antirheumatic drugs (DMARDs), and biological agents (Watanabe et al., 2022[83]). Although these treatments can alleviate symptoms and slow disease progression, they have significant limitations. They provide short-term symptomatic relief, but prolonged use is associated with adverse effects, including gastrointestinal inflammation, cardiovascular risk, immunosuppression, and osteoporosis-related risks. DMARDs and biologics are equally effective but take weeks or even months until their effectiveness is reached, and are accompanied by high costs in most patients, immunogenic reactions, and increased risk for infections (Santiago-Garcia et al., 2023[64]). Moreover, the systemic administration of anti-inflammatory medications often leads to inadequate concentrations of the drug at the joint inflammation site, simultaneously subjecting healthy tissues to superfluous exposure to these treatments (Wirth et al., 2024[88]). This nonspecific distribution diminishes therapeutic efficacy and elevates the likelihood of adverse effects, thereby highlighting the necessity for more localized and targeted delivery strategies (Akram et al., 2021[4]).

### 1.3 Rationale for using dexamethasone

Dexamethasone (DEX) is a highly active synthetic glucocorticoid whose anti-inflammatory and immunosuppressive action is achieved by altering pro-inflammatory gene expression, reducing leukocyte migration, and inhibiting cytokine synthesis (Lorscheider et al., 2019[43]; Huebner et al., 2014[30]). It is widely used in the therapy of acute and chronic inflammatory diseases, including arthritis. However, its use in a systemically administered form is limited by potential side effects, including adrenal suppression, hyperglycemia, osteoporosis, and increased susceptibility to infections (Tarasova et al., 2025[73]). To address such issues, targeted delivery systems, particularly drug formulations involving liposomes, have attracted significant interest.

Liposomes are lipid bilayers formed as spherical vesicles that are able not only to encapsulate hydrophilic but also lipophilic drugs, thus augmenting their bioavailability and pharmacokinetics (Tarasova et al., 2025[73]). DEX can be encapsulated in liposomes such that it is directed specifically towards inflamed tissues, either by passive targeting mechanisms like the enhanced permeability and retention (EPR) effect or active targeting by means of surface ligands (Zamanian et al., 2025[92]). DEX-Lips is a novel method for enabling extended and localized drug delivery in arthritic sites that might enable a reduction in systemic toxicity while maximizing therapeutic effect (Zamanian et al., 2025[92]; Pourmadadi et al., 2025[57]). Such a method is in line with the ultimate goals of precision medicine, augmenting effectiveness in minimizing side effects, and might represent a significant step forward in arthritic treatment.

## 2. Dexamethasone in Arthritis Management

### 2.1 Mechanism of anti-inflammatory action

**Figure 2:**
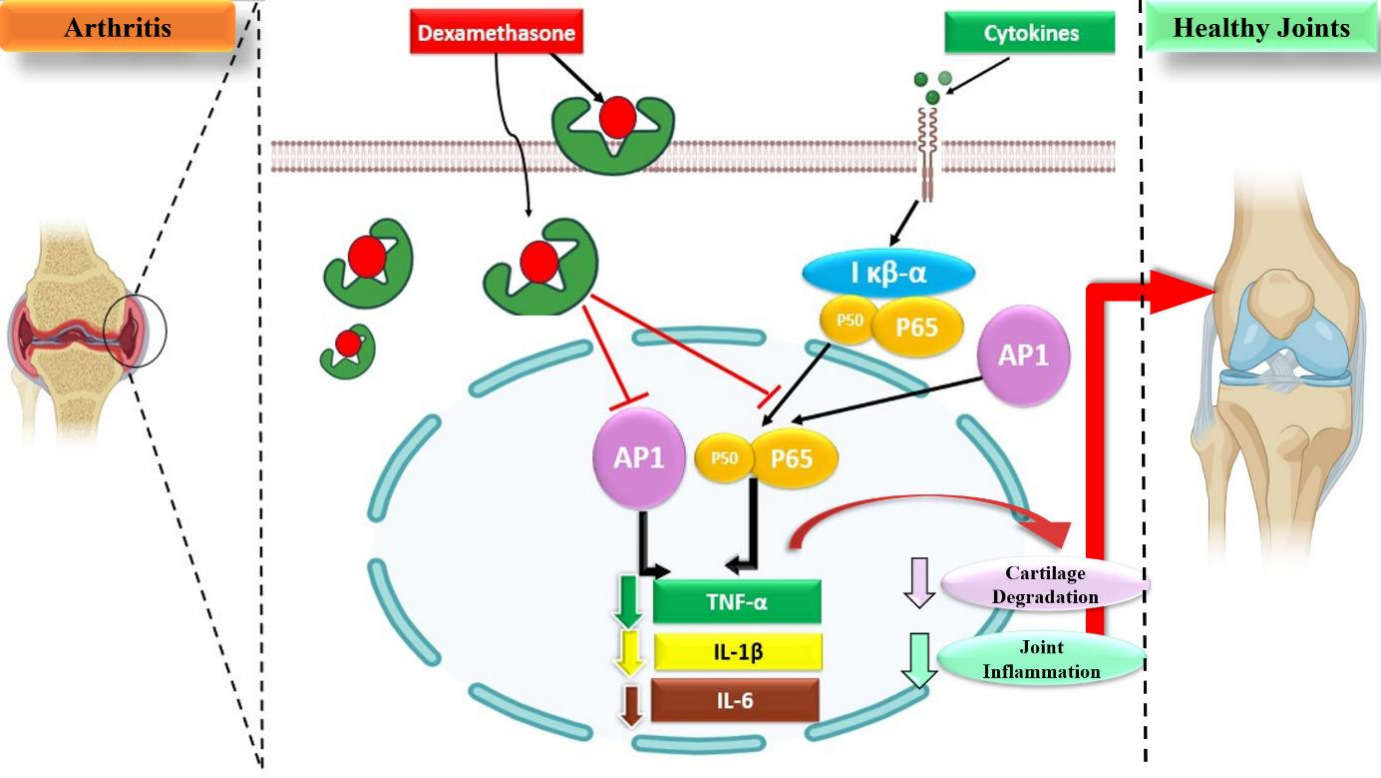
Mechanism of anti-inflammatory action of dexamethasone in arthritis: The diagram depicts the intracellular anti-inflammatory action of dexamethasone in arthritic joint cells. Upon cellular entry, dexamethasone binds to cytoplasmic GR to form a ligand-receptor complex. This complex is transported to the nucleus, where it suppresses pro-inflammatory transcription factors, NF-κB and AP-1, thereby downregulating pro-inflammatory cytokines TNF-α, IL-1β, and IL-6. This cascade ultimately leads to reduced inflammation, less immune cell infiltration, and prevention of cartilage destruction in arthritic joints.

DEX is a potent synthetic glucocorticoid commonly used for its anti-inflammatory and immunosuppressive functions in several chronic inflammatory diseases such as arthritis. Its main method of action involves binding to intracellular glucocorticoid receptors (GRs), which then travel to the nucleus to modulate gene expression (Abraham et al., 2006[1]). Such action results in upregulation of anti-inflammatory proteins like lipocortin-1, in combination with pro-inflammatory mediator downregulation, such as interleukins (IL-1β, IL-6), tumor necrosis factor-alpha (TNF-α), and cyclooxygenase-2 (COX-2) (Seo and Priefer, 2020[65]). Furthermore, DEX inhibits activation of nuclear factor-kappa B (NF-κB), which is an essential transcription factor in inflammatory cytokine and chemokine production. In the context of RA, DEX attenuates leukocyte infiltration within the synovial membrane and suppresses synovial hyperplasia. In other OA, it mitigates cartilaginous degeneration by downregulating MMP expression. Through such multiple mechanisms of action, DEX allows for rapid, effective control of localized as well as generalized inflammation in arthritis (Figure 2(Fig. 2)) (Reichardt et al., 2021[62]).

### 2.2 Pharmacokinetics and therapeutic potential

DEX possesses favorable pharmacokinetic features, making it an effective agent in treating inflammatory disorders. It shows high oral bioavailability, which is set at about 80-90 %, and shows a wide plasma half-life ranging between 3-6 hours, in combination with a variable biological half-life ranging between 36-54 hours for its slow glucocorticoid receptor occupancy (Queckenberg et al., 2011[60]; Bashir and Acosta, 2020[11]). Such features allow for longer-acting anti-inflammatory effects, thus reducing dosage frequency needed for clinical benefits. Once absorbed, DEX is widely distributed throughout the body, including in synovial fluid, where it carries out its pharmacological activity. Its metabolism occurs in the liver via cytochrome P450 enzymes, while its main route of excretion is through urine (Madamsetty et al., 2022[45]). Clinically, DEX is used in both acute and chronic conditions for symptom relief in arthritis, specifically in flare-ups. DEX can be administered in its oral, intravenous, intramuscular, or intra-articular forms, depending on the intensity of inflammation and clinical goals (Yao et al., 2025[91]). Due to its strong and broad immunosuppressive activity, DEX is a valuable resource for treating autoimmune arthritis, particularly in conditions where rapid symptom relief is needed or when patients respond insufficiently to DMARDs or biologic agents (Yao et al., 2025[91]).

### 2.3 Epigenetic mechanism and epigenetic reprogramming

**Figure 3:**
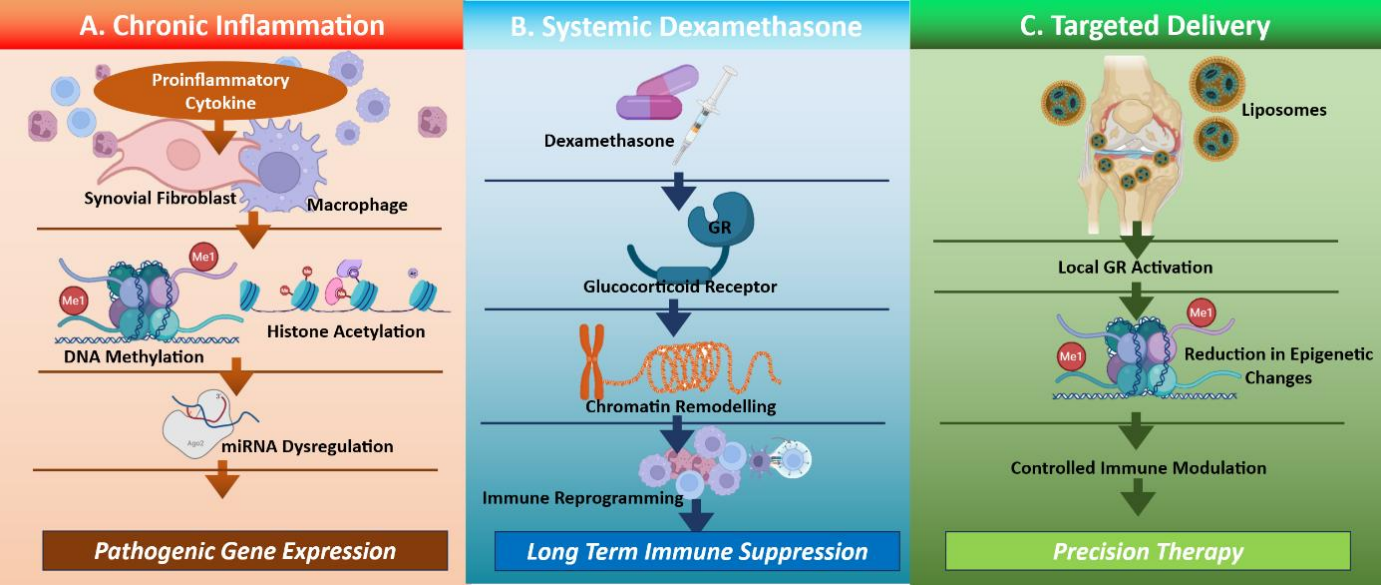
Epigenetic consequences and DEX therapy in RA

Recent evidence suggests that epigenetic processes, heritable changes in gene expression that are not caused by changes in the DNA sequence, are key players in the development and maintenance of chronic inflammatory arthritis. Epigenetic control of gene expression includes DNA methylation, histone modifications, and non-coding RNA expression, and these mechanisms affect key effector cells such as synovial fibroblasts (Figure 3(Fig. 3)) (Araki and Mimura, 2016[8]). In RA, synovial fibroblasts have an activated phenotype with enhanced proliferation, invasiveness, and the capacity to continuously produce pro-inflammatory mediators. These pathogenic changes in synovial fibroblasts in RA have been associated with differential DNA methylation in gene promoters and enhancers. This results in changes in chromatin structure without alterations in the DNA sequence. Aberrant DNA methylation may either suppress anti-inflammatory genes or activate pro-inflammatory genes (Nemtsova et al., 2019[51]). Histone modifications, such as acetylation, methylation, and phosphorylation, also play a role in regulating chromatin structure and gene expression. For example, histone acetylation levels can be adjusted to either open chromatin to activate gene expression or close chromatin to suppress gene expression. Imbalances in histone modification have also been associated with the chronic expression of inflammatory gene networks in RA (Krishna Priya et al., 2025[39]). Furthermore, the regulation of inflammatory and matrix remodeling genes by non-coding RNAs, especially microRNAs, adds another level of post-transcriptional regulation that reinforces disease-specific expression profiles. Together, these epigenetic regulators form a self-perpetuating network that reinforces the pathogenic phenotypes in the synovial and immune cells (Krishna Priya et al., 2025[39]).

This epigenetic landscape not only perpetuates chronic inflammation through a mechanism independent of genetic sequence variation but also creates a mechanistic link through which environmental stimuli, such as prolonged exposure to inflammatory cytokines and therapeutic agents, can induce lasting alterations in gene expression regulation.

Epigenetic regulation is important for immune cell differentiation, activation, and functional plasticity. DNA methylation, histone tail modifications, and chromatin structure are important in regulating gene expression in immune cells. DNA methylation of CpG sites or histone acetylation/methylation status can result in sustained changes in gene expression. This can affect immune responses and memory (Zatterale et al., 2022[93]). This is because the glucocorticoid signaling pathway can affect this process by recruiting chromatin-remodeling enzymes to GR-binding sites, reorganizing the immune cell epigenome. This effect is long-lasting, even after the drug is no longer present, effectively reprogramming the immune response (Wiencke et al., 2022[86]).

DEX affects arthritis through epigenetic reprogramming; for instance, the evidence shows two distinct patterns. First, prenatal DEX exposure paradoxically increases susceptibility to arthritis through epigenetic mechanisms: prenatal DEX exposure has been shown to affect immune regulation by epigenetically reprogramming the glucocorticoid receptor gene (NR3C1). DEX exposure during pregnancy can lead to changes in DNA methylation in promoter regions of NR3C1, especially in exon 1 variants, resulting in decreased expression of the glucocorticoid receptor (GR) in immune cells. As GR mediates the anti-inflammatory actions of glucocorticoids by regulating immune-modulating gene transcription, including NF-κB-regulated genes such as TNF-α and IL-6, decreased GR expression can lead to decreased glucocorticoid receptor signaling and reduced regulation of these pathways. As a result, these changes can enhance immune activation of pro-inflammatory pathways, increasing susceptibility to autoimmune and arthritis-like inflammatory diseases. DNA methylation is an epigenetic change that is stable and can decrease gene transcription without altering the DNA sequence, which allows prenatal exposure to DEX to have long-lasting effects on immune regulation (Sun et al., 2016[72]). In line with the above findings, another study on prenatal exposure to DEX has shown that it influences immune regulation through epigenetic reprogramming mechanisms. Experimental studies have shown that prenatal exposure to DEX can result in DNA methylation of the glucocorticoid receptor gene (NR3C1), leading to reduced GR expression in the immune system. The GR signaling pathway is known to inhibit inflammatory responses by suppressing the transcription of pro-inflammatory cytokines such as TNF-α and IL-6 by the transcription factor NF-κB. Reduced expression of the GR receptor, therefore, leads to increased inflammatory responses. These changes, therefore, suggest that prenatal exposure to DEX increases the risk of inflammatory responses, such as those observed in arthritis, through epigenetic reprogramming mechanisms mediated by stable DNA methylation of immune-related genes (Achuthan, 2022[2]).

### 2.4 Challenges with systemic administration

Though clinically effective, the systemic administration of DEX in arthritis is fraught with serious issues, mostly related to side effects consequent upon its non-specific distribution and extended systemic exposure. Its chronic use is attributed to a plethora of side effects, such as glucocorticoid-induced osteoporosis, adrenal suppression, hyperglycemia, atrophied muscles, hypertension, mood disorders, as well as susceptibility to infections due to immunosuppression (Wang et al., 2025[76]; Li et al., 2018[41]). Systemic administration is also incapable of providing effective high concentrations at sites of inflammation, for instance, at synovial joints, thus rendering local therapeutic effectiveness suboptimal. Frequent dosing to sustain drug levels at the therapeutic level further enhances susceptibility to systemic toxicity (Almutairi et al., 2021[5]). Besides, in conditions such as RA, in which inflammation is continuous in incidence and the multiplicity of joints involved is high, determining specific sites for targeting is not easy with conventional systemic administration. These issues emphasize the critical need for site-specific, sustained-release drug delivery systems capable of delivering DEX specifically to inflamed joints while reducing its systemic absorption, thereby enhancing efficacy and safety in the long-term management of arthritis.

## 3. Liposomes as Drug Delivery Vehicles

### 3.1 Structure and types of liposomes

**Figure 4:**
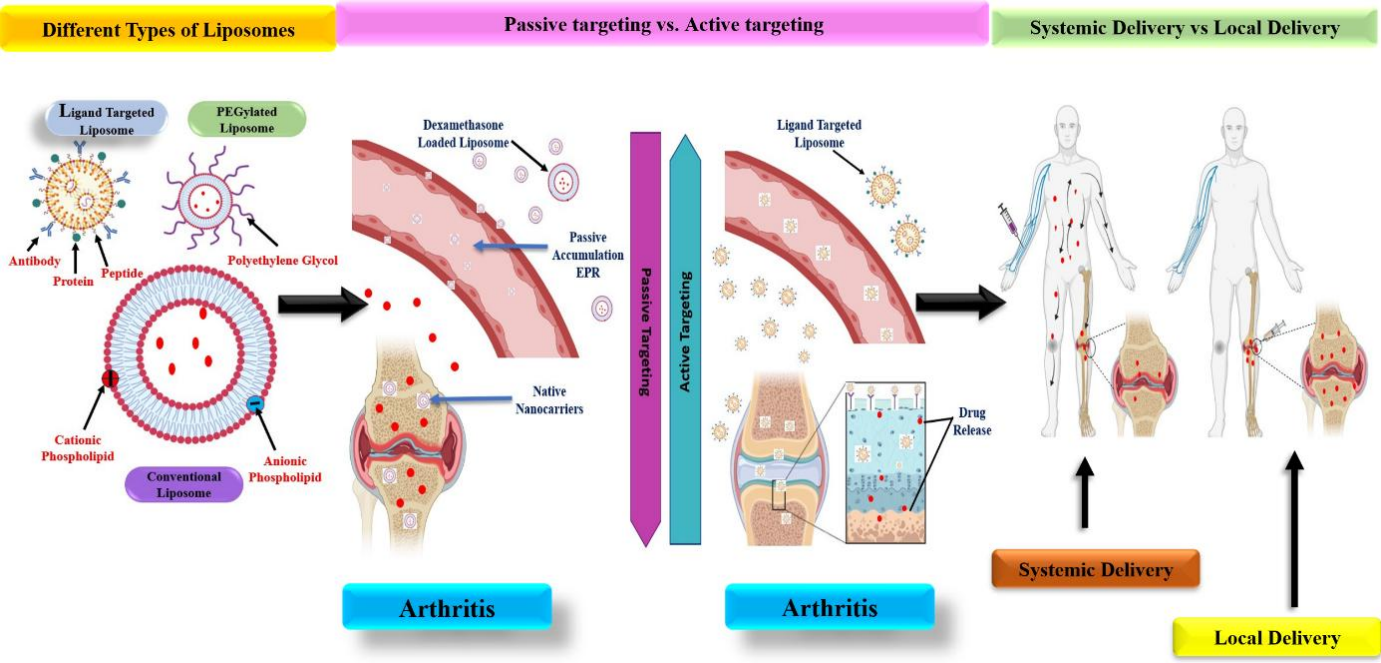
Structure and targeting mechanisms of liposomal drug delivery systems

Liposomes are submicellar vesicular carriers composed of a single or multiple layers of phospholipids surrounding an aqueous core, which makes them effective carriers for hydrophilic as well as for lipophilic drugs (Figure 4(Fig. 4)) (Kapoor et al., 2025[35]). Because their structure is similar to that of a cell membrane, liposomes are efficient at interacting with cells and biological membranes. Depending on size, number of bilayers, and composition, liposomes are divided into small unilamellar vesicles (SUVs, 20-100 nm in diameter), large unilamellar vesicles (LUVs, 100-1000 nm in diameter), and multilamellar vesicles (MLVs, above 500 nm in diameter) (Andra et al., 2022[7]). In addition to size variation, surface modifications that attach polyethylene glycol (PEG) chains (PEGylation) are commonly used to enhance stability and extend residence time in the bloodstream by suppressing RES recognition and clearance (Ren et al., 2019[63]). Moreover, liposomes may be prepared with pH- or thermosensitive membranes, or with membranes that respond to stimuli, enabling controlled drug release in response to certain environmental stimuli, such as in inflamed joints involved in arthritis.

### 3.2 Advantages of liposomal drug delivery

Liposomal formulations confer several noteworthy advantages over conventional drug delivery approaches, most notably in therapies for chronic inflammatory disorders like arthritis (van Alem et al., 2021[75]). Initially, liposomes can encapsulate and protect drugs from early metabolic degradation in the bloodstream, thereby enhancing their stability and bioavailability. Second, formulations allow for sustained and controlled drug release, which can prolong pharmacologic effects while reducing dosing regimen frequencies (Zhang et al., 2025[98]). Such is especially beneficial for drugs like DEX, which require careful dosing to depress systemic toxicity. Thirdly, liposomes enhance the pharmacokinetics and biodistribution of encapsulated drugs, thereby favoring a higher concentration at disease sites, such as inflamed synovial tissues. Favored localization at such sites minimizes off-site effects while reducing exposure for healthy tissues (Zhang et al., 2025[98]). Moreover, biocompatibility and biodegradability characterize liposomes, enabling repeated administration. As an application for arthritis therapy, drug delivery by liposomes has proved effective in supplementing anti-inflammatory activity while reducing unwanted effects to provide a more patient-friendly and effective therapeutic modality (Zhao et al., 2022[99]; Zhang et al., 2025[98]).

### 3.3 Passive vs. active targeting strategies

Passive targeting exploits the increased permeability and retention (EPR) effect, a phenomenon frequently observed in inflammation or neoplasia, in which leaky endothelial vessels and impaired lymphatic clearance permit nanoparticles, such as liposomes, to accumulate (Attia et al., 2019[9]). In an arthritic model, for instance, an inflamed joint exhibit enhanced vascular permeability, which facilitates passive sequestration within the synovium. Passive targeting remains sensitive to compromise by variables such as liposome size, charge, or circulatory half-life, any of which must be carefully optimized for maximum effectiveness. On the other hand, active targeting involves modification of the liposome surface with ligands such as antibodies, peptides, or small molecules that specifically bind to overexpressed receptors on target cells (Ferreira-Silva et al., 2021[22]; Zhu et al., 2022[100]). In arthritis therapy, it is possible to design ligands for selective targeting towards macrophages, synoviocytes, or endothelial cells expressing markers like folate receptor-β, integrins, or CD44. Active targeting can significantly improve cell uptake of liposomes and drug delivery efficiency in target tissue while compensating for the limitations inherent in passive accumulation (Ferreira-Silva et al., 2021[22]). When combined with stimuli-responsive release mechanisms, active targeting allows for extremely specific on-demand drug release in response to an inflammatory milieu, such that it significantly improves therapeutic outcomes in arthritis therapy (Chen et al., 2017[17]).

## 4. DEX-Lips for Arthritis Therapy

DEX is a potent glucocorticoid widely used to control inflammation in arthritis (Reichardt et al., 2021[62]). Despite its efficacy, clinical application is hampered by rapid clearance, non-specific distribution, and dose-dependent adverse effects, including osteoporosis, metabolic dysfunction, and immune suppression (Reichardt et al., 2021[62]). Liposomal encapsulation offers a promising strategy to address these limitations by prolonging circulation, enhancing drug accumulation in inflamed joints, and reducing systemic toxicity. Over time, successive DEX-Lips designs have been developed to meet evolving therapeutic needs in arthritis management (Table 1(Tab. 1); References in Table 1: Benne et al., 2024[12]; Chang et al., 2021[15]; Kulikov et al., 2021[40]; Meka et al., 2019[47]; Rauchhaus et al., 2009[61]; Wang et al., 2021[79]; Wang et al., 2025[77]; Zhang et al., 2025[97]; Zhang et al., 2025[98]; Zhao et al., 2022[99]).

### 4.1 Rheumatoid arthritis (RA)

DEX remains central in RA therapy, though pharmacokinetic limitations and safety issues restrict its use. Liposomal strategies have evolved to address these barriers: conventional liposomes enhanced stability and sustained release, PEGylated systems prolonged circulation with passive targeting (Anderson et al., 2010[6]), and ligand-modified liposomes enabled active delivery to synovial macrophages (Meka et al., 2019[47]). More recently, stimuli-responsive liposomes achieve localized, controlled release in inflamed joints. This progressive refinement offers improved efficacy and reduced systemic toxicity, advancing DEX-based RA therapy.

#### Conventional liposomes for passive targeting

Conventional DEX-liposomes show strong efficacy in experimental arthritis, with PEG-free design minimizing immunogenicity and hypersensitivity risks (Avnir et al., 2008[10]).

**Figure 5:**
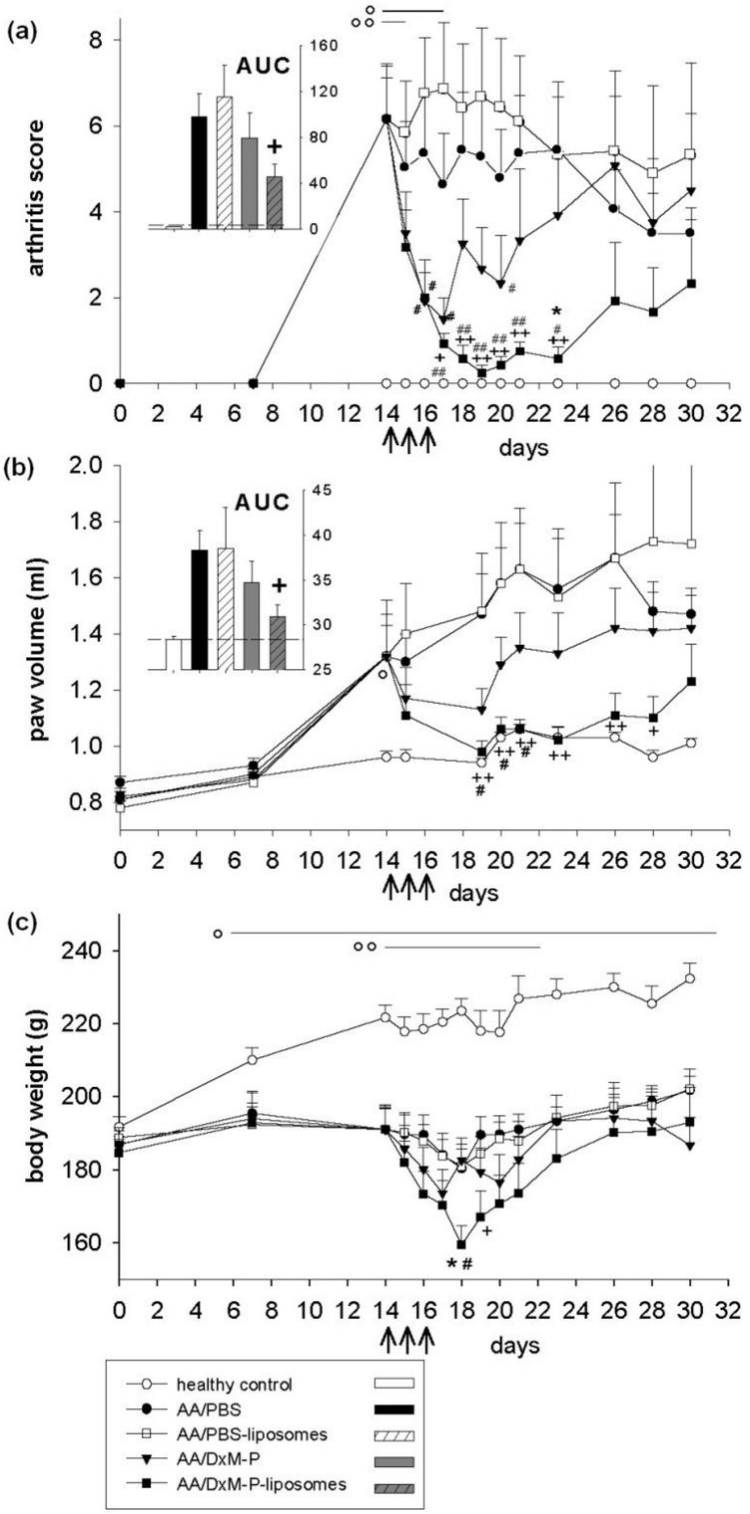
Clinical effects of free and DEX-Lips phosphate (DxM-P) in adjuvant arthritis: Rats received PBS-liposomes, free DxM-P (3 × 1 mg/kg; Days 14-16), or liposomal DxM-P (3 × 1 mg/kg; Days 14-16). Shown are changes in arthritis score (a), paw volume (b), and body weight (c). Inserts depict the area under the curve (AUC, Day 0-30) for arthritis score and paw volume; dashed line indicates healthy control levels. Adapted with permission from Anderson et al. (2010) under CC BY 2.0

Anderson and the team of researchers (Anderson et al., 2010[6]) evaluated the efficacy of intravenous (i.v.) DEX-Lips phosphate (DxM-P) compared with free DxM-P in rats with established adjuvant arthritis (AA). Treatment with non-PEGylated liposomes produced significant and long-lasting suppression of inflammation and joint swelling at 3-10 times lower doses than the free glucocorticoid. A short 3-day course not only reduced arthritis scores but also prevented nearly 80 % of flare-ups after therapy withdrawal, demonstrating a depot-like effect. Encapsulation enabled a dose reduction of 3-10-fold, with drug levels remaining significantly higher in plasma, synovial membrane, spleen, and liver for at least 48 hours post-injection. Short-term i.v. treatment with liposomal DxM-P (3 × 1 mg/kg on Days 14-16 of AA) nearly normalized arthritis scores and paw volume (Figure 5a, b(Fig. 5); Reference in Figure 5: Anderson et al., 2010[6]).

By Day 28, two weeks after initiation, the formulation maintained a 54 % reduction in arthritis score and a 73 % reduction in paw swelling. In contrast, the same dosage of free DxM-P produced only transient improvements, with rapid rebound after treatment cessation. PBS-liposomes were ineffective and occasionally worsened symptoms. The area under the curve (AUC) further demonstrated the superiority of liposomal encapsulation: arthritis score AUC was reduced by 54 % (versus 19 % with free drug), and paw swelling AUC by 74 % (versus 36 % with free drug; Figure 5a, b(Fig. 5)). Despite a temporary drop in body weight (Figure 5c(Fig. 5)), treatment was well tolerated, and animals quickly regained normal activity (Anderson et al., 2010[6]).

These findings were consistently reproduced in independent experiments, validating the robustness of the results. The formulation selectively accumulated in inflamed joints, spleen, and liver, where it acted as a localized drug depot. This site-specific retention prolonged therapeutic activity, limited systemic exposure, preserved joint integrity by reducing cartilage and bone erosion, and suppressed pro-inflammatory cytokines such as IL-1β and IL-6.

Rauchhaus and colleagues developed liposomes using DPPC, DPPG, and cholesterol (50:10:40 mol%) via lipid film extrusion for RA and related inflammatory disorders. Encapsulation of DEX phosphate (DXM-P) resulted in a more sustained anti-inflammatory effect than the free drug. Remarkably, a single 4 mg/kg dose of liposomal DXM-P provided therapeutic benefit with fewer glucocorticoid side effects, even after drug clearance by day 2, suggesting an improved therapeutic window (Rauchhaus et al., 2009[61]). Although conventional liposomes lack PEGylated carriers' extended circulation, they balance efficacy and safety, avoid PEG-associated immune responses, and still achieve notable anti-inflammatory activity.

#### PEGylated (stealth/long-circulating) liposomes

Conventional liposomes are attractive drug carriers but face two major limitations. Their circulation time in the blood is short due to rapid clearance by the reticuloendothelial system (RES), and their structural stability under physiological conditions is often insufficient, resulting in premature drug leakage. Surface modification with polyethylene glycol (PEG) provides a hydrophilic “stealth” layer that minimizes RES recognition and prolongs systemic circulation, thereby improving drug bioavailability (Patel et al., 2024[55]).

To improve membrane robustness, lipid polymerization within the bilayer has been developed, enhancing structural integrity and reducing premature leakage. More recently, the combination of PEGylation with polymerized lipid systems has yielded polymerized stealth liposomes, which integrate prolonged circulation with improved stability (Makharadze et al., 2025[46]; Hofkens et al., 2011[27]). These advanced carriers show promise for achieving sustained therapeutic activity and reducing off-target effects, making them particularly relevant for the treatment of chronic inflammatory diseases such as RA (Ferreira-Silva et al., 2021[22]).

In arthritic animal models, such PEGylated liposomes carrying anti-inflammatory agents like DEX not only demonstrated prolonged presence in the circulation but also preferentially accumulated in inflamed joints, suppressing pro-inflammatory cytokines such as TNF-α and IL-1β and significantly reducing joint swelling and disease progression (Prasad et al., 2015[58]).

Beyond PEGylation, researchers have explored (Wang et al., 2020[78]) polymer-stabilized stealth liposomes to improve stability and therapeutic durability. Wang et al. developed polymerized stealth DEX Liposomes using 1,2-bis(10,12-tricosadiynoyl)-sn-glycero-3-phosphocholine (DC8,9PC) and DSPE-PEG2000 via thin-film hydration. In this system, DC8,9PC molecules were UV-crosslinked within the bilayer, enhancing structural integrity, while surface PEG chains provided a stealth coating to extend circulation. Polymerized liposomes exhibited excellent stability, prolonged circulation, and efficient cellular uptake with minimal toxicity.

In arthritic rat models, these polymerized liposomes showed superior therapeutic efficacy compared to free dexamethasone, with greater reductions in joint inflammation, paw swelling, and pro-inflammatory cytokines such as TNF-α and IL-1β. Overall, these findings suggest improved therapeutic outcomes due to enhanced stability, sustained drug release, and better accumulation at inflamed sites. However, translation requires further validation of safety, reproducibility, and long-term outcomes (Wang et al., 2020[78]).

#### Localized DEX-Lips delivery for intra-articular and transdermal approaches

In RA, intravenous delivery of nanocarriers often results in drug loss and poor targeting, while intra-articular (IA) delivery improves local action and reduces systemic side effects but suffers from rapid clearance and frequent injections (Song et al., 2022[70]). Liposomal encapsulation of DEX overcomes these limitations by protecting the drug from enzymatic degradation, prolonging IA half-life, and reducing injection frequency, thereby lowering the risks of joint infection and tissue damage. Early studies demonstrated that DEX-Lips (dexamethasone palmitate) achieved superior joint retention and anti-inflammatory efficacy compared to microcrystalline steroids, with larger vesicles (~750 nm vs. 160 nm) further enhancing synovial retention. Notably, DEX-Lips avoided systemic cortisol suppression and showed a safer profile than free DEX in RA models, with minimal effects on body weight, blood glucose, and hematological parameters (Jia et al., 2018[34]). These findings underscore its potential as an effective strategy to improve IA retention while minimizing systemic exposure.

Kulikov et al. developed DEX-Lips (vesicle size ~86 nm; drug concentration 2.2 mg/mL) for intra-articular use in a rat arthritis model. Compared with free DEX solution at the same dose, the liposomal formulation produced greater improvements in both macroscopic and microscopic indicators of joint inflammation, demonstrating superior therapeutic efficacy for arthritis management (Kulikov et al., 2021[40]). Chondroitin sulfate (ChS) protects cartilage by preventing IL-1β-induced apoptosis and promoting regeneration and repair of cartilage tissue. Combining anti-inflammatory action with cartilage repair provides an effective approach for RA therapy. To achieve this, DEX liposomes incorporating ChS and gelatin offer significant therapeutic potential (Siddiqui et al., 2024[69]; Wang et al., 2020[82]).

Zhang and colleagues developed a hyaluronic acid-modified liposomal depot (HA-Lipo@G/D) encapsulating a dexamethasone-loaded nanogel, enabling CD44-mediated targeting and enhanced retention in inflamed joints (Zhang et al., 2025[98]). In arthritis models, it showed superior efficacy over free drug, significantly reducing inflammation, joint swelling, and pro-inflammatory cytokines while improving cartilage preservation and IL-10 levels. Overall, this system combines targeted delivery with sustained release for improved therapeutic outcomes, though challenges in clinical translation and long-term safety remain (Zhang et al., 2025[98]).

Wang and team of researchers developed sialic acid-decorated DEX-Lips palmitate (DP-SALs) to target peripheral blood neutrophils, which naturally migrate to inflamed joints in RA. They tested three sizes (300, 150, and 75 nm) and found that particle size strongly influenced uptake, migration, and therapeutic effect. Sialic acid helped neutrophils selectively carry DP-SALs to inflamed sites. Large liposomes had higher drug loading but impaired neutrophil function, while small DP-SALs preserved neutrophil activity, localized efficiently in arthritic joints, and showed superior efficacy. In rats, small DP-SALs reduced swelling, joint damage, and pro-inflammatory cytokines (TNF-α, IL-1β), offering the best targeted and long-lasting RA therapy (Wang et al., 2021[79]).

**Figure 6:**
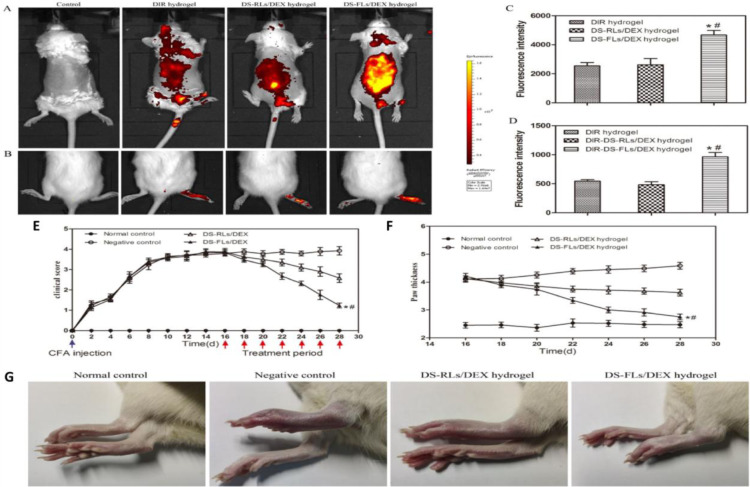
In vivo distribution and therapeutic evaluation of DS-RLs/DEX and DS-FLs/DEX hydrogels in AIA rats: (A) Skin localization of DIR-tagged DS-RLs/DEX and DS-FLs/DEX hydrogels observed via live imaging; (B) Joint targeting and accumulation assessed using fluorescence imaging; (C, D) Quantitative interpretation of imaging results; (E) Arthritis severity scores and (F) average paw swelling measurements; (G) Representative macroscopic images of the hind limbs. Modified with permission from Zhao et al. (2022) under CC BY 4.0

For localized therapy in mono- or oligo-articular RA, intra-articular liposomal injections sustain joint drug levels but are limited by short action and systemic effects. Transdermal delivery offers safer, steady dosing, though hindered by skin barriers. Zhao and team of investigators (Zhao et al., 2022[99]) synthesized a transdermal dextran sulfate (DS)-modified (DEX)-loaded flexible liposome hydrogel (DS-FLs/DEX hydrogel) and systematically verified its transdermal effectiveness, macrophage targeting, and anti-inflammatory property. The DS-FLs/DEX hydrogel showed excellent biocompatibility, long-term drug release, and targeted uptake by lipopolysaccharide (LPS)-activated macrophages. In vivo imaging demonstrated significantly higher skin accumulation, with fluorescence intensity reaching 4,665 ± 324 for DS-FLs/DEX, compared to 2,623 ± 431 for DS-RLs/DEX and 2,545 ± 226 for free drug, indicating ~1.7-1.8-fold higher permeability (Figure 6A-D(Fig. 6); Reference in Figure 6: Zhao et al., 2022[99]). Therapeutically, both hydrogels reduced arthritis severity, as seen by decreased clinical scores and paw thickness (Figure 6E, F(Fig. 6)). DS-FLs/DEX showed superior efficacy, with paw thickness nearly returning to normal levels, consistent with improved clinical scores. Macroscopic observations of hind limbs also confirmed reduced inflammation (Figure 6G(Fig. 6)). Overall, the enhanced effect of DS-FLs/DEX is attributed to its higher deformability and improved transdermal delivery to inflamed joints (Zhao et al., 2022[99]).

#### Peptide-targeted liposomal formulation

Peptide-targeted liposomal systems have gained attention for their ability to enhance drug selectivity and efficacy in RA. One of the most widely studied approaches uses the RGD (arginine-glycine-aspartic acid) peptide, a well-characterized integrin-binding motif, to functionalize PEGylated liposomes for targeting αvβ3 integrins on angiogenic endothelial cells within inflamed joints. These RGD-modified liposomes demonstrated efficient binding and internalization by vascular endothelial cells in vitro and preferential accumulation at inflamed sites in vivo, as confirmed by intravital microscopy. Importantly, encapsulation of DEX phosphate in RGD-PEG liposomes resulted in sustained suppression of arthritis progression in the adjuvant-induced arthritis model, providing superior outcomes compared to free drug and non-targeted liposomes (Koning et al., 2006[38]).

Building on this concept, Meka and the team of investigators engineered a peptide-directed liposomal delivery system for DEX (DEX-LPs-Target) for enhanced therapeutic efficacy in RA. A peptide sequence, CKPFDRALC (designated ART-2), demonstrated selective accumulation in arthritic rat joints and strong affinity for endothelial cells (Meka et al., 2019[47]).

Therapeutic efficacy was tested in the collagen-induced arthritis (CIA) model in mice, wherein peptide-directed DEX-LPs showed significantly greater anti-arthritic activity compared with free DEX and control, non-targeted liposomes. Together, RGD- and ART-2-modified liposomes illustrate the promise of peptide-guided targeting in RA, offering improved drug delivery to diseased tissues while reducing systemic toxicity (Meka et al., 2019[47]).

However, translation to clinical settings requires addressing potential drawbacks, including the immunogenicity of peptide ligands, the stability of peptide-lipid conjugation, and the scalability of production. Long-term safety and efficacy studies remain essential to validate their promise as next-generation RA therapeutics.

An alternative strategy to deliver DEX to inflamed joints is to use nanoparticles that interact with albumin. Albumin is abundant, biocompatible, has a long half-life, and naturally accumulates in inflamed tissues. Instead of directly formulating DEX with albumin, Wang et al. (2024) formulated liposomes modified with an albumin-binding domain (ABD), a 46-residue peptide derived from streptococcal protein G. This allows the liposomes to capture endogenous albumin in circulation, extending blood half-life and enhancing drug accumulation at inflamed sites. ABD functionalization improved albumin binding, cellular uptake, and in vivo targeting. In rats with adjuvant-induced arthritis, ABD-Lip/DEX accumulated at inflamed joints, reduced swelling, and lowered TNF-α and IL-1β levels, without notable toxicity or hematological side effects.

#### Hyalurosomes co-loaded with DEX and luteolin

Single-drug therapies often inadequately control RA progression. While DEX effectively suppresses inflammation, its long-term use is constrained by systemic toxicity. Luteolin (LUT), a natural flavonoid with antioxidant and NF-κB inhibitory activity, provides complementary benefits. However, its poor solubility and low bioavailability hinder therapeutic potential, highlighting the need for advanced delivery strategies.

To address these challenges, a dual-drug delivery platform based on hyaluronic acid (HA)-modified liposomes, termed hyalurosomes, was engineered by Zewail and colleagues (Zewail et al., 2025[94]) to co-encapsulate DEX and LUT. This strategy integrates the potent anti-inflammatory effects of DEX with the antioxidant and NF-κB-modulating activity of LUT. Surface modification with HA improves accumulation in inflamed synovial tissue via CD44 receptor targeting while prolonging intra-articular retention. Sustained co-release of both agents enhances local efficacy and minimizes systemic exposure (Zewail et al., 2025[94]). The formulation demonstrated high drug encapsulation efficiency (~93 % for DEX and ~81 % for LUT) and controlled release, with less than 50 % of either drug released within 24 h. In adjuvant-induced arthritis rats, DEX-LUT hyalurosomes significantly reduced inflammatory and oxidative markers, including TNF-α, IL-1, MMP-3, and MDA, while enhancing NRF2 expression. These biochemical outcomes confirmed the synergistic suppression of inflammatory and oxidative pathways. Joint diameters were recorded on days 0, 3, 7, and 14. Notably, the DEX-LUT hyalurosome group showed no statistical difference from the negative control, suggesting near-complete restoration of joint function. Morphological assessment at day 14 revealed that while blank hyalurosomes and single-drug liposomes improved joint condition, the dual-drug formulation provided the most pronounced recovery, with outcomes almost identical to healthy controls.

Co-delivery of DEX and LUT via hyalurosomes represents a rational therapeutic approach for RA. By combining anti-inflammatory and antioxidant activities with HA-mediated targeting and sustained release, this system achieves superior efficacy and safety compared to single-agent liposomal formulations. Future studies should focus on optimizing dosing strategies, evaluating long-term safety, and exploring translation to human clinical settings. Moreover, extending this co-delivery concept to other natural compounds or DMARDs may broaden therapeutic potential and establish dual-drug liposomes as a versatile platform for next-generation RA therapy (Zewail et al., 2025[94]).

#### Multifunctional DEX-loaded ginsenoside compound K liposomes

Cholesterol is traditionally incorporated into liposomes to increase bilayer rigidity and extend circulation. However, prolonged exposure can activate complement protein C3, leading to immune clearance and inflammation (Inglut et al., 2020[33]). These limitations have motivated exploration of safer substitutes that preserve structural stability while adding therapeutic benefits. One promising candidate is ginsenoside compound K (CK), a rare ginseng metabolite with a sterol-like structure. CK demonstrates anti-inflammatory, anticancer, and immunomodulatory activity and can stabilize lipid bilayers similar to cholesterol. In RA, it reduces synovial inflammation, inhibits T-cell activation, and suppresses glycolysis through NF-κB/HIF-1α signaling. Nonetheless, CK suffers from poor solubility, low bioavailability, and limited accumulation in joints, restricting its clinical use (Hong et al., 2019[28]).

**Figure 7:**
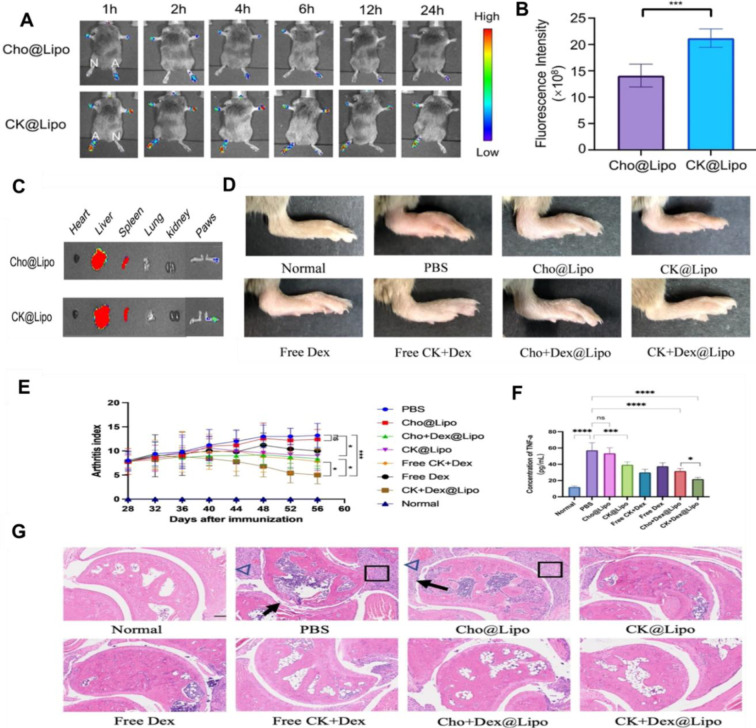
In vivo targeting and therapeutic effects of liposomes in CIA mice: (A) Fluorescence imaging of normal and inflamed paws after intravenous administration; (B) Quantified joint fluorescence intensity; (C) Biodistribution at 48 h post-injection; (D) Representative arthritic paw images; (E) Arthritis index across groups; (F) Serum TNF-α levels; (G) H&E-stained joint sections showing cartilage erosion (→), neovascularization (△), and synovial hyperplasia (☐). Adapted with permission from (Zhang et al., 2025) under CC BY 4.0

RA pathology involves synovial inflammation, angiogenesis, and cartilage damage, producing vascular hyperpermeability akin to the tumor EPR effect. Leveraging this feature, Zhang and colleagues (Zhang et al., 2025[97]) designed ginsenoside-based liposomes using CK as both a cholesterol substitute and therapeutic agent, co-loaded with DEX for synergistic benefit. The resulting CK + DEX@Lipo system was evaluated against conventional cholesterol liposomes (Cho@Lipo) in vitro and in vivo. Figure 7(Fig. 7) (Reference in Figure 7: Zhang et al., 2025[97]) illustrates the biodistribution. In vivo fluorescence imaging showed that DiR-labeled liposomes preferentially accumulated in inflamed paws of CIA mice, but not in healthy joints (Figure 7A(Fig. 7)). Quantitative analysis revealed ~1.5-fold higher fluorescence intensity for CK@Lipo compared to Cho@Lipo (Figure 7B(Fig. 7)). At 48 hours, CK@Lipo demonstrated enhanced joint localization with reduced off-target organ uptake (Figure 7C(Fig. 7)), likely mediated by ginsenoside-binding receptors. Therapeutic outcomes confirmed CK's advantage. As shown in Figure 7D and E(Fig. 7), PBS-treated mice exhibited severe swelling and progression of arthritis, whereas Cho@Lipo provided negligible benefit. CK@Lipo significantly lowered swelling and arthritis indices, confirming CK's dual stabilizing and therapeutic function. Free DEX and DEX@Lipo improved symptoms, but the most profound improvement occurred with CK + DEX@Lipo, which nearly restored normal joint morphology. Cytokine analysis (Figure 7F(Fig. 7)) showed that CK@Lipo reduced TNF-α, while CK + DEX@Lipo produced the strongest suppression. Histopathology (Figure 7G(Fig. 7)) revealed that only the CK + DEX formulation prevented cartilage erosion, synovial hyperplasia, and immune infiltration, closely resembling healthy tissue. Mechanistically, CK integrates into liposomal bilayers to confer stability and therapeutic efficacy, while DEX broadly suppresses cytokine production. Together, they synergize to control RA progression. Replacing cholesterol with CK represents a paradigm shift in liposome design, transforming a structural component into a multifunctional therapeutic element. Although clinical translation requires overcoming manufacturing, pharmacokinetic, and safety hurdles, CK-based liposomes hold promise as next-generation platforms for RA and other immune, metabolic, and oncologic diseases.

#### Mesenchymal stem cell-based biomimetic liposome (MSCsome)

Cell membrane-based biomimetic carriers offer biocompatibility, immune evasion, and targeted delivery, showing therapeutic potential in cancer, infections, and autoimmune diseases (Xiao et al., 2022[89]; Liu et al., 2023[42]). Among these, mesenchymal stem cell (MSC) membranes are especially attractive due to low immunogenicity, easy expansion, and innate homing to inflamed tissues (Castro Nava et al., 2023[14]). Their surface proteins, including chemokine receptors (CXCR4, CXCR1, CCR2) and adhesion molecules (VCAM-1, VLA-4, LFA-1), enhance migration (Cui et al., 2024[18]).

Building on this, Ma et al. developed MSC-membrane-coated liposomes encapsulating DEX (DEX-MSCsomes) for RA. In a CIA mouse model, DEX-MSCsomes showed superior anti-inflammatory effects, reduced swelling, and preserved cartilage compared with free DEX and conventional DEX-liposomes (Ma et al., 2024[44]).

DEX-MSC-somes show promising targeting and therapeutic effects in RA, but key challenges include standardizing membrane isolation, scalable production, and ensuring long-term safety and stability. Optimizing drug loading, release, and pharmacokinetics remains crucial. Future directions involve co-delivery, patient-specific membranes, stimuli-responsive release, and AI-guided monitoring, with potential to expand applications to other autoimmune and inflammatory disorders.

#### Stimuli-triggered liposomal drug delivery in RA

Stimuli-responsive liposomes represent next-generation strategies for RA therapy. Unlike conventional carriers, they remain intact under physiological conditions but undergo controlled activation in response to pathological cues, thereby enabling localized drug release and reducing systemic toxicity (Zhang et al., 2022[96]). In RA joints, acidic pH (6.4-6.6), high levels of reactive oxygen species (ROS), and overexpression of matrix metalloproteinases (MMP-1, MMP-3, MMP-13) provide exploitable triggers. Liposomes engineered with pH-sensitive lipids or ROS-cleavable linkers, such as thioketals and boronates, enable selective release. Recent designs integrate stimulus-responsiveness with active targeting or depot strategies to further enhance therapeutic precision (Kondo et al., 2023[37]).

Song and a team of researchers introduced a dual-functional liposome (DEX@FA-ROS-Lips) incorporating ROS-cleavable lipids and folic acid ligands for macrophage targeting. This system enabled receptor-mediated uptake followed by ROS-triggered drug release, effectively suppressing the iRhom2/TNF-α/BAFF axis and reducing inflammation in arthritis models, while maintaining good hemocompatibility (Song et al., 2021[71]). Similarly, Ni and colleagues developed ROS-sensitive, folate-conjugated nanoparticles targeting both macrophages and fibroblast-like synoviocytes, achieving potent suppression of synovial hyperplasia (Ni et al., 2020[52]). Together, these approaches demonstrate the therapeutic promise of multifunctional, stimuli-responsive liposomes. Despite challenges in stability, scalability, and clinical validation, these “smart” carriers hold strong potential to advance corticosteroid-based RA therapy.

#### hPGK4-DEX liposomes for antigen-specific therapy

Antigen-specific immunotherapy represents a highly promising strategy for autoimmune diseases, as it enables selective suppression of pathogenic immune responses while preserving normal immune function (Page et al., 2021[54]). Central to this approach are tolerogenic dendritic cells (tolDCs), which suppress pro-inflammatory Th1 and Th17 subsets while promoting the expansion of regulatory T cells (Tregs, Tr1s) (Domogalla et al., 2017[20]). This immunological reprogramming restores tolerance by reducing cytokines such as IL-12 and TNF-α and enhancing IL-10 and TGF-β. Importantly, tolDCs can be engineered with disease-specific antigens, offering precise modulation without broad immunosuppression. While early clinical studies in RA have produced encouraging findings (Willekens et al., 2019[87]; Phillips et al., 2019[56]), their broader application is constrained by high costs, variability, and the need for specialized production facilities.

Nanoparticles, particularly liposomes, are being investigated as scalable, cost-effective alternatives. Liposomes are biocompatible vesicles that can encapsulate antigens and drugs while directing immune responses. For example, anionic DSPG (2-distearoyl-sn-glycero-3-phosphoglycerol) liposomes enhance antigen uptake by dendritic cells and stimulate Treg activation, yielding therapeutic benefits in preclinical autoimmune models (Benne et al., 2018[13]). DEX, a potent glucocorticoid inducer of tolDCs, is effective but limited by poor solubility, rapid clearance, and systemic toxicity. However, its clinical application is hampered by poor solubility, rapid systemic clearance, and significant side effects (Madamsetty et al., 2022[45]).

To address these issues, Benne and a group of investigators (Benne et al., 2024[12]) designed a novel system where DEX was conjugated to human proteoglycan (hPG) via a lysine tetramer (K4) and biodegradable spacer, then encapsulated within DSPG liposomes (hPGK4-DEX liposomes) (Benne et al., 2024[12]). In arthritic mice, this approach reduced autoantibody levels, expanded Tregs, and boosted IL-10 production in inflamed joints, thereby inducing antigen-specific tolerance.

### 4.2 Osteoarthritis

Osteoarthritis is the most prevalent chronic joint disorder, affecting more than 300 million individuals worldwide and representing a major cause of disability in the aging population. The disease is characterized by progressive cartilage degeneration, subchondral bone remodeling, osteophyte formation, and low-grade synovial inflammation, which together contribute to chronic pain and functional impairment (Hunter and Eyles, 2022[32]).

Despite its high prevalence and socioeconomic burden, current therapeutic approaches remain largely palliative. Conventional pharmacological strategies, including NSAIDs, intra-articular corticosteroid injections, and hyaluronic acid, provide symptomatic relief but fail to halt or reverse structural disease progression. Moreover, repeated systemic corticosteroid administration, such as DEX, is associated with significant adverse effects, including immunosuppression, metabolic dysfunction, and systemic toxicity (Kloppenburg and Berenbaum, 2020[36]).

Liposomal formulations of DEX have emerged as a promising strategy in this context. Encapsulation of DEX within liposomes prolongs intra-articular drug retention, reduces dosing frequency, and enhances local anti-inflammatory effects, thereby potentially improving patient outcomes in OA. By enabling targeted drug release in inflamed synovium and cartilage, liposomal systems may reduce systemic toxicity and offer a disease-modifying advantage over conventional corticosteroid therapies (Kloppenburg and Berenbaum, 2020[36]; Mitsou and Klein, 2025[48]).

Preclinical studies support this approach, demonstrating that DEX-Lips nanoparticles can alleviate joint inflammation, protect cartilage, and sustain therapeutic efficacy compared with free-drug formulations.

#### Intra-articular DEX liposomes (DEX-Lips)

In OA, intra-articular (IA) corticosteroid injections provide only short-lived pain relief and may worsen cartilage degeneration with repeated administration. DEX-Lips formulations were developed to overcome these drawbacks by extending drug retention within the joint and enabling sustained release. Following IA injection, liposomes are efficiently taken up by synovial macrophages, key mediators of glucocorticoid action in inflamed joints, which promotes localized delivery and prolonged anti-inflammatory activity. DEX-Lips has shown superior intra-articular persistence, reduced joint swelling, protection against cartilage erosion, and lower levels of inflammatory cytokines compared with free DEX solution. These features reduce dosing frequency while enhancing chondroprotection, highlighting DEX-Lips as a promising long-acting and safer alternative for OA management (Kulikov et al., 2021[40]).

Recent studies highlight that synovial macrophage polarization plays a central role in OA progression. In OA, macrophages accumulate within the synovium, where they can adopt either a pro-inflammatory M1 phenotype or an anti-inflammatory M2 phenotype depending on the local microenvironment. This balance not only drives synovial inflammation but also influences pain modulation, as M1 and M2 macrophages contribute differently to pain initiation and resolution (Zhang et al., 2020[95]).

To address this, Teng and colleagues (Teng et al., 2023[74]) evaluated intra-articular dexamethasone-loaded liposomes (DEX-Lipo) for osteoarthritis (OA), showing improved local retention and reduced systemic exposure, along with macrophage repolarization toward the anti-inflammatory M2 phenotype. In OA models, DEX-Lipo effectively reduced synovial inflammation, preserved cartilage integrity, and suppressed macrophage infiltration and pro-inflammatory cytokines (IL-1β, TNF-α), demonstrating strong therapeutic potential (Teng et al., 2023[74]).

Formulations with slower drug release have shown superior joint protection by sustaining therapeutic effects longer than faster-releasing systems. Intra-articular DEX-Lips extended the treatment window compared to free DEX (around 10 vs. 6 days), but its effect was still less durable than polymer conjugates or crosslinked micelles (Quan et al., 2014[59]).

This limitation is linked to the structural fragility of liposomes, where drug leakage or vesicle destabilization may occur during circulation. In contrast, conjugated and crosslinked carriers retain the drug more securely. These findings highlight the importance of improving liposomal stability in the joint environment, where intra-articular delivery helps preserve vesicle integrity and prolong local activity.

Preclinical and translational studies further support this approach. For instance, TLC599, a DEX-Lips sodium phosphate formulation, showed extended joint residence of up to 120 days in canine models and has progressed to phase III clinical trials (Hunter et al., 2022[31]). Similarly, Lipotalon® (DEX palmitate, 4 mg/mL) remains the only approved liposomal intra-articular therapy available on the German market (Mitsou and Klein, 2025[48]). These examples demonstrate the clinical potential of DEX-Lips in the treatment of OA. However, most current formulations are relatively simple, focusing on sustained release without fully addressing challenges such as mechanical joint stress, heterogeneous intra-articular distribution, or macrophage-targeted delivery.

#### Dual-drug encapsulation in liposomes

Liposomes are highly suitable for co-delivery systems since their bilayer structure can simultaneously encapsulate both water-soluble and lipid-soluble agents. This enables the incorporation of different therapeutic classes within a single carrier. In OA, where conventional oral NSAIDs and intra-articular corticosteroids are limited by toxicity, liposomal formulations of diclofenac and DEX have been developed. Using hyaluronan (HA-BAL) or collagen-modified (COL-BAL) bioadhesive liposomes, both drugs, individually or in combination, showed high loading efficiency (> 80 %), controlled release over 1-3 days, and maintained biological activity. In OA rat models, a single intra-articular dose reduced joint inflammation for up to 17 days, with HA-BAL outperforming COL-BAL. The dual-drug HA-BAL formulation was most effective, decreasing inflammation volume to 12.9 % of baseline.

Hyaluronic acid (HA) is a naturally occurring gel-like substance that supports cartilage and bone development, while also reducing inflammation and pain from joint injury or degeneration. Because of these properties, HA injections are widely used for OA pain management (Huang et al., 2019[29]).

Building on this, Chang et al. developed an HA-based liposomal system co-loaded with diclofenac (DIC) and DEX for localized OA therapy. The formulation included both hydrophilic (DEX sodium phosphate) and hydrophobic (DEX base) forms of DEX along with DIC, allowing fast therapeutic action and sustained release for over 7 days. In OA mouse models, the dual-drug liposomes significantly reduced inflammation and leukocyte infiltration with only minor tissue reactions (Chang et al., 2021[15]).

**Figure 8:**
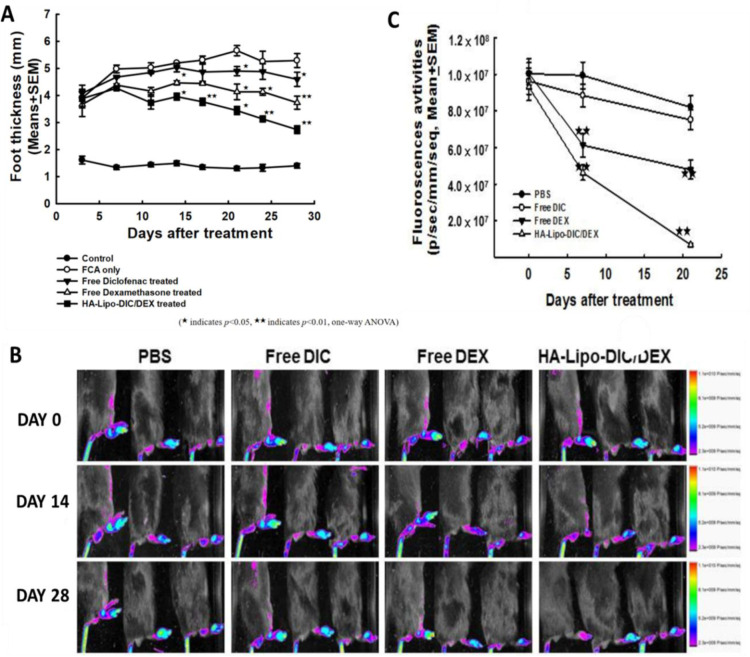
(A) Anti-arthritic efficacy and in vivo imaging of HA-Lipo-DIC/DEX in C57BL/6 mice: Arthritis was induced using FCA, followed by a single intra-articular dose of free DIC, free DEX, or HA-Lipo-DIC/DEX from day 8, with the opposite paw as control. In vivo fluorescence imaging using NE680 was performed to assess inflammation. (B) Representative near-infrared images showing drug distribution in paws. (C) Quantitative fluorescence analysis indicating reduced inflammation, with the HA-Lipo-DIC/DEX group. Adapted with permission from Chang et al. (2021) under CC BY 4.0

In vivo results demonstrated that HA-Lipo-DIC/DEX was more effective in reducing paw swelling than the individual drugs. After 28 days, paw thickness decreased to 2.74 mm with the dual-drug system, compared to 4.60 mm for DIC and 3.73 mm for DEX alone (Figure 8A(Fig. 8); Reference in Figure 8: Chang et al., 2021[15]), confirming its superior anti-inflammatory efficacy. Further evaluation using in vivo fluorescence imaging showed that, following 7 days without treatment and 14 days of therapy, the treated group displayed the lowest fluorescence intensity in the affected paw (Figure 8B(Fig. 8), C, p < 0.001). This indicates a significant reduction in neutrophil elastase activity and overall inflammation (Chang et al., 2021[15]).

However, for optimal outcomes, both drugs should ideally act on the same target cells (such as macrophages). If their targets differ, it may lead to uneven therapeutic effects. In such cases, strategies like controlled or staged drug release become important. Thus, while dual-drug liposomes are promising, their success depends on proper drug selection, controlled release design, and precise targeting of diseased tissue.

#### Cartilage protection via lubricative liposomes

In OA, cartilage deterioration arises not only from chronic inflammation but also from impaired lubrication at the articular surface, which increases friction and accelerates mechanical wear. Recent research highlights the potential of large, empty liposomes as biomimetic lubricants when administered intra-articularly. These vesicles coat cartilage surfaces, reduce shear stress, and limit further degeneration independently of drug delivery. For instance, multilamellar liposomes have been shown to restore boundary lubrication in OA rat models, mimicking the role of natural synovial phospholipids and reducing cartilage damage.

Normal cartilage lubrication relies on hyaluronic acid, lubricin, and phospholipids, which act synergistically to maintain smooth articulation. In OA, depletion of these components disrupts joint lubrication. Liposomes, with amphiphilic bilayers, can restore function by forming hydration shells, boundary films, and filling surface irregularities.

**Figure 9:**
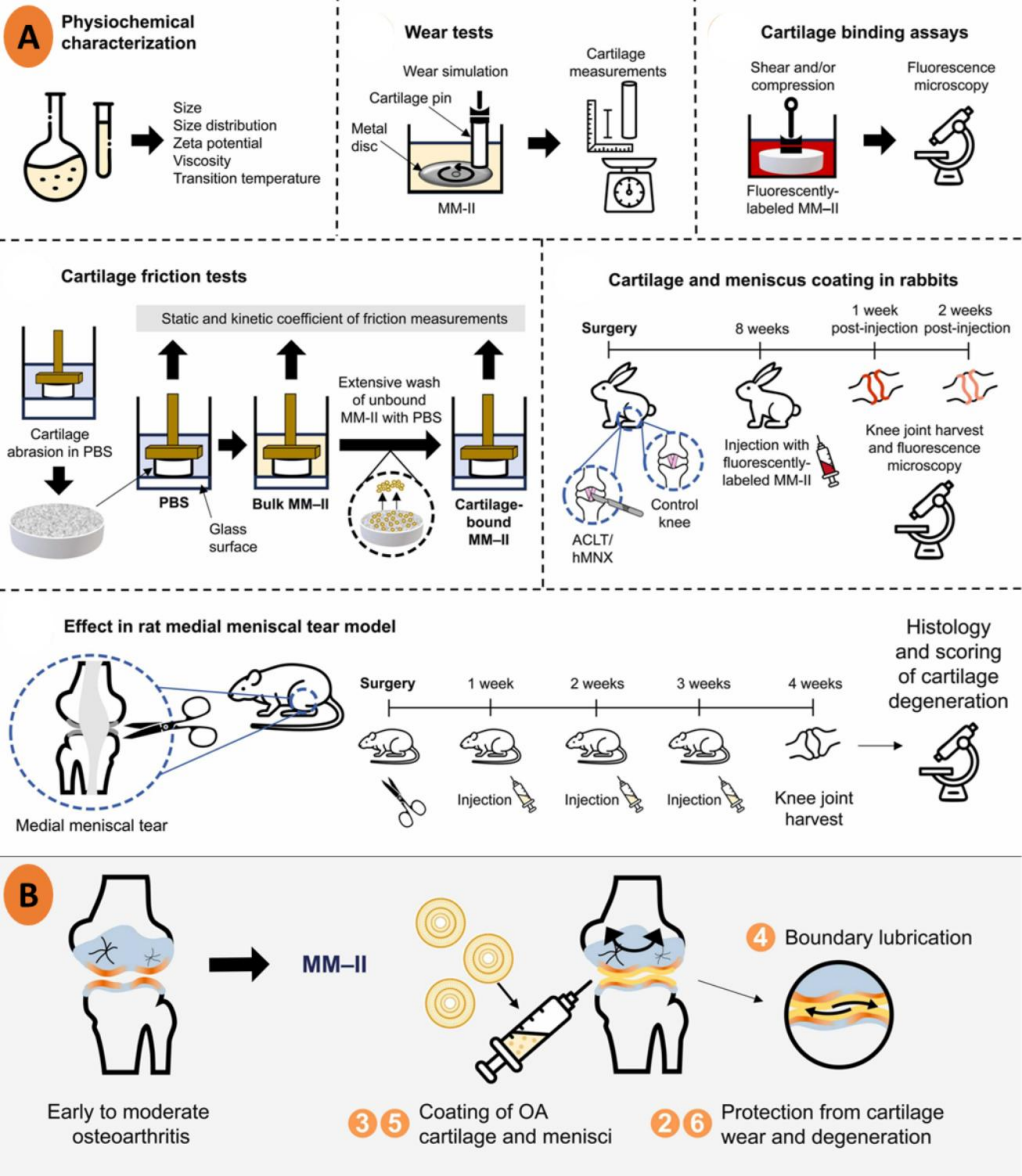
(A) Schematic overview of MM-II studies assays and (B) proposed mechanism of action. The physicochemical characteristics of MM-II were first evaluated, followed by assessment of its cartilage-binding capacity and protective effects against wear using binding and friction assays. Lubrication performance was measured on cartilage discs after removing unbound liposomes. In vivo, the capacity of MM-II to coat cartilage and menisci and to prevent cartilage degeneration was examined in both rat and rabbit models of osteoarthritis. Adapted with permission from Wechsler et al. (2025) under CC BY 4.0

Wechsler and colleagues (Wechsler et al., 2025[84]) developed MM-II, a liposomal formulation designed to function within OA-relevant temperature ranges. To investigate its mechanism and structural effects, cartilage coating was assessed using fluorescently labeled MM-II under compressive and non-compressive conditions. The lubricative function was quantified in cartilage-on-glass friction assays and pin-on-disc wear models, while intra-articular distribution was examined in healthy and OA rabbit knees. Structural protection was further evaluated in a rat OA model, comparing MM-II with individual DMPC or DPPC liposomes, their combination, and vehicle controls. Results demonstrated that MM-II preferentially adhered to cartilage under load, forming a stable boundary layer that reduced friction and wear. In rats, MM-II decreased tibial cartilage degeneration by 53 % and limited mononuclear cell infiltration. These findings suggest that MM-II provides chondroprotection primarily through boundary lubrication, with additional synovial modulation contributing to clinical pain relief, highlighting its potential as a biomimetic lipid-based OA therapy. Figure 9(Fig. 9) (Reference in Figure 9: Wechsler et al., 2025[84]) illustrates the study design (Figure 9A(Fig. 9)) and the proposed mechanism of action (Figure 9B(Fig. 9)) (Wechsler et al., 2025[84]).

Although drug-loaded liposomes, such as DEX formulations, emphasize anti-inflammatory effects, their contribution to lubrication remains underexplored. Optimizing liposomal size, lipid composition, and hydration properties could maximize both tribological performance and therapeutic efficacy in OA.

## 5. Challenges and Limitations

### 5.1 Drug leakage and stability issues

A major technical challenge involving liposomal drug delivery involves the problems of leakage and insufficient stability, both at the level of storage as well as upon administration (Giannopoulos-Dimitriou et al., 2024[26]). Liposomes are naturally sensitive to physical and chemical stressors, such as temperature variations, pH changes, and oxidation, which can compromise membrane integrity and trigger early drug release. This issue is particularly strong in the case of small, hydrophilic drugs like DEX, which can leak out of the liposomal core unless retained adequately (Nsairat et al., 2022[53]; Giannopoulos-Dimitriou et al., 2024[26]).

Further, lipid bilayers are vulnerable to hydrolysis or oxidation when exposed over time, which can negatively impact their structural integrity and lead to variable dosing. Several formulation approaches, such as PEGylation, cholesterol inclusion, or the use of saturated phospholipids, can improve stability; nonetheless, none eliminate leakage problems. Furthermore, ensuring an extended shelf life while retaining encapsulation efficiency and drug efficacy remains a major formulation hurdle, particularly when scaling up for commercial production purposes (Shah et al., 2025[66]).

### 5.2 Immunogenicity and toxicity concerns

Although commonly perceived as biocompatible, liposomes' interactions with the immune system can pose a range of risks. Immunogenic responses are influenced by parameters such as liposome composition, size, charge, and administration route. Cationic liposomes are found to be more likely to stimulate pro-inflammatory reactions and cause cell toxicity (Weiss et al., 2023[85]). Moreover, repeated administration of PEGylated liposomes can result in anti-PEG antibodies developing in a so-called “accelerated blood clearance” (ABC) effect, leading to rapid clearance of successive doses with a consequent loss in therapeutic efficacy (Shiraishi and Yokoyama, 2024[68]).

Liposomes are further susceptible to sequestration by mononuclear phagocyte system (MPS) cells in specific organs such as spleen and liver, with possible off-target sequestration leading to systemic toxicity (Inglut et al., 2020[33]). Moreover, if the drug, in its encapsulated form, leaks into the systemic circulation, it can persist in its undesirable activity, thereby negating the advantage of targeted delivery. Hence, an in-depth biosafety evaluation for both the therapeutic cargo and the vehicle in a liposomal form is necessary.

### 5.3 Regulatory and translational barriers

Preparation of liposomes requires advanced equipment and precise control of variables such as particle size, polydispersity, entrapment efficiency, and sterility. Reproducible consistency at an expanded level of such parameters is a substantial issue in clinical-grade production. Furthermore, full characterization as well as validation studies for toxicity profiling, evaluation for immunogenicity, and long-term stability are demanded by regulatory agencies like the FDA and EMA for liposomal drug products (Wang and Grainger, 2022[80]).

Liposomal drug formulations are considered, in some cases, drug-device combinations relative to small molecules; such combinations are subject to increased regulatory scrutiny (Wang et al., 2023[81]).

Apart from this, an insufficient standard methodology for pharmacokinetic as well as biodistribution evaluation in human beings, combined with efficacy, continues to hinder their incorporation into clinical use (Eugster and Luciani, 2025[21]).

Overall, expense, regulatory issues, and translational obstacles remain significant roadblocks to broader clinical applications of DEX-Lips in arthritic treatment.

## 6. Future Perspectives

### 6.1 Advances in liposome technology

New technologies in liposomes are significantly altering the therapeutic direction for drug delivery in chronic inflammatory disease, including arthritis. One key area for innovation is creating stimuli-responsive liposomes that release their drug payloads upon encountering specific stimuli, such as shifts in pH, temperature, redox gradients, or enzyme activity-parameters found in inflamed joints. Moreover, ultrasound- or magnetic-field-responsive liposomes are increasingly studied in an attempt to permit greater spatial and temporal control over drug delivery (Filipczak et al., 2020[23]).

Advances in lipid chemistry, including the incorporation of novel artificial lipids and stabilizers, are increasing membrane stability while minimizing early drug leakage. Further, the use of microfluidic device construction enables greater control over liposome size, composition, and drug encapsulation efficiency, thereby enabling scalable and reproducible production processes. These technological advances hold promise for greater therapeutic precision, less systemic toxicity, and greater clinical adoption for DEX-Lips formulations (Chauhan and Gupta, 2020[16]; Ajeeshkumar et al., 2021[3]).

### 6.2 Personalized and targeted therapy potential

The future direction of arthritis therapy is increasingly toward personalized medicine (Sharma and Bluett, 2024[67]), for which liposomal formulations are particularly well-suited to enable. By functionalizing liposomes with surface ligands or antibodies that recognize patient-specific biomarkers or receptors on immune cells, therapies can be tailored to the patient's disease phenotype and molecular features. As an illustration, for RA patients, liposomes may be customized to specifically target activated macrophages or synovial fibroblasts expressing surface markers such as CD44 or folate receptor-β. Through such an active targeting method, drug concentration at disease sites would be maximized while minimizing the effect on normal tissues (Yao et al., 2025[91]). Indeed, integrating individual inflammatory profiles or genetic predispositions into treatment approaches might guide the selection among different liposomal formulations, dosing schedules, or administration intervals (van Alem et al., 2021[75]). With the greater availability of genomic and proteomic technologies, there is greater feasibility and clinical utility in using liposomal drug carriers to deliver highly personalized disease-modifying therapies.

### 6.3 Integration with imaging and diagnostics

An exciting frontier in the field of liposomes is the development of theranostic liposomes in which both diagnostic and therapeutic functions are combined in a single nanocarrier (Xing et al., 2016[90]). These multi-functional liposomes can carry a variety of contents, ranging from pharmaceuticals (e.g., DEX) to imaging agents, including fluorescent dyes, MRI contrast agents, and radioactive tracers. Such integration allows clinicians to have real-time visualization of drug distribution, assessment of treatment efficacy, and responsive adjustment of dosing. When it comes to arthritis therapies, theranostic liposomes are used for the detection of inflamed joints by imaging modalities, assessment of disease severity, and confirmation of successful delivery of the drug agent (Yao et al., 2025[91]). Using non-invasive modalities such as PET, SPECT, or MRI in combination with liposomal preparations not only enhances therapeutic outcomes but also improves patient safety and compliance. As diagnostics and nanomedicine come together, the field of theranostics can radically change arthritis therapies by allowing real-time image-guided personalized treatment strategies (Deprez et al., 2022[19]).

## 7. Conclusion

Arthritis remains a disabling disease that imposes significant clinical and societal impacts, drawing urgent attention towards developing more specific and effective treatment modalities. Though DEX is a very effective anti-inflammatory drug, its clinical use is limited by severe side effects as well as non-specific biodistribution. The use of liposomal encapsulation offers a promising approach by enhancing the targeted delivery, bioavailability, and safety profile of DEX in the treatment of arthritis. Despite formulation hurdles and regulatory clearance issues, continued innovation in liposome design, biomarker-directed targeting, and theranostic use is considerably diversifying the therapeutic repertoire. Integration of nanotechnology, personalized medicine, and real-time diagnostics holds immense promise for developing the next wave of arthritis therapies with a focus on safety, intelligence, and patient-oriented modalities. Continued inter-disciplinary research and investment in translational platforms shall hold the key towards realizing the full promise of DEX-Lips in routine clinical use.

## Notes

Anil Pareek, Swapnil Sharma (Department of Pharmacy, Banasthali Vidyapith, Banasthali-304022, Rajasthan, India; E-mail: skspharmacology@gmail.com) and Devesh U. Kapoor (Dr. Dayaram Patel Pharmacy College, Bardoli-394601, Gujarat, India; Phone: +91-7874223242, E-mail: dev7200@gmail.com and deveshkapoor@dppc.ac.in) contributed equally as corresponding author.

## Declaration

### Acknowledgments

The author Shadma Wahab extends their appreciation to the Deanship of Research and Graduate Studies at King Khalid University for funding them through a Small Group Research Project under grant number (RGP2/133/47).

### Funding

Shadma Wahab received funding from Deanship of Research and Graduate Studies at King Khalid University through a Small group Research Project under grant number (RGP2/133/47).

### Conflict of interest

The authors declare that they have no known competing financial interests or personal relationships that could have appeared to influence the work reported in this paper.

### Artificial Intelligence (AI) - assisted technology

AI models such as Grammarly and QuillBot have been used for the refinement of English language in this manuscript.

### Author contribution

Kajal Kumari: Wrote the manuscript; Anil Pareek: Wrote the manuscript; Shadma Wahab: Reviewed the manuscript, funding acquisition; Sachin Sharma: Software, prepared the diagrams; Vipin Saini: Wrote the manuscript, software; Swapnil Sharma: Reviewed the manuscript, supervision; Devesh U. Kapoor: Conceptualization, reviewed the manuscript, project administration

### Data availability

The authors confirm that the data supporting the findings of this study are available within the articles referenced.

### Ethics approval and consent to participate

Not applicable.

## Figures and Tables

**Table 1:**
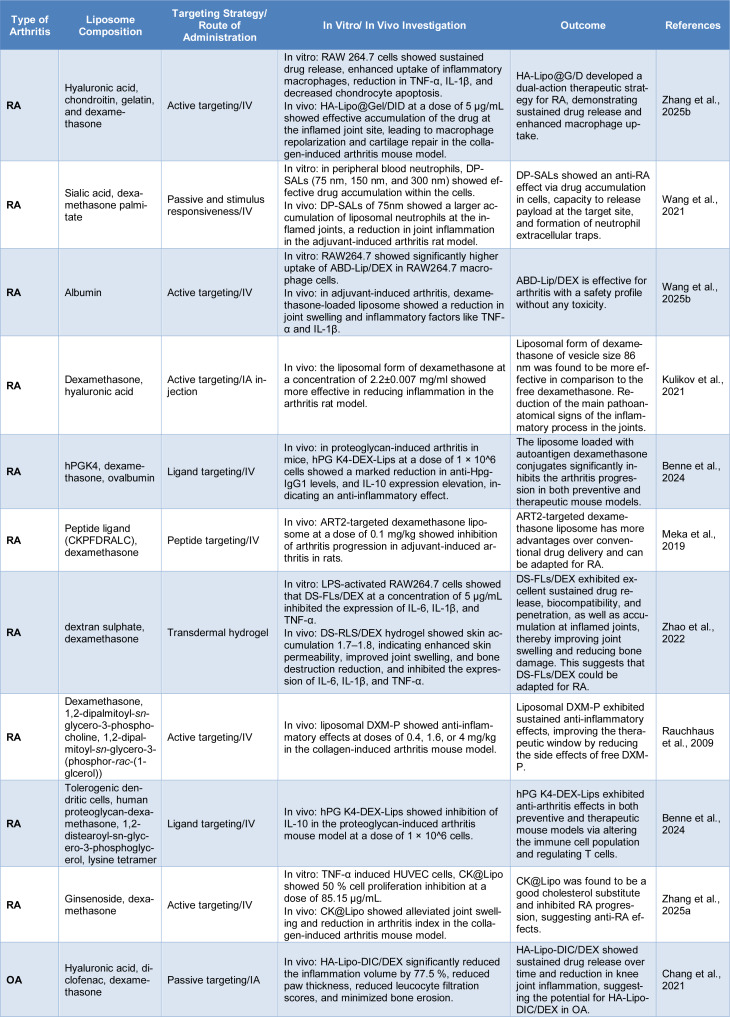
Summary of liposomal dexamethasone for arthritis therapy

**Figure 1:**
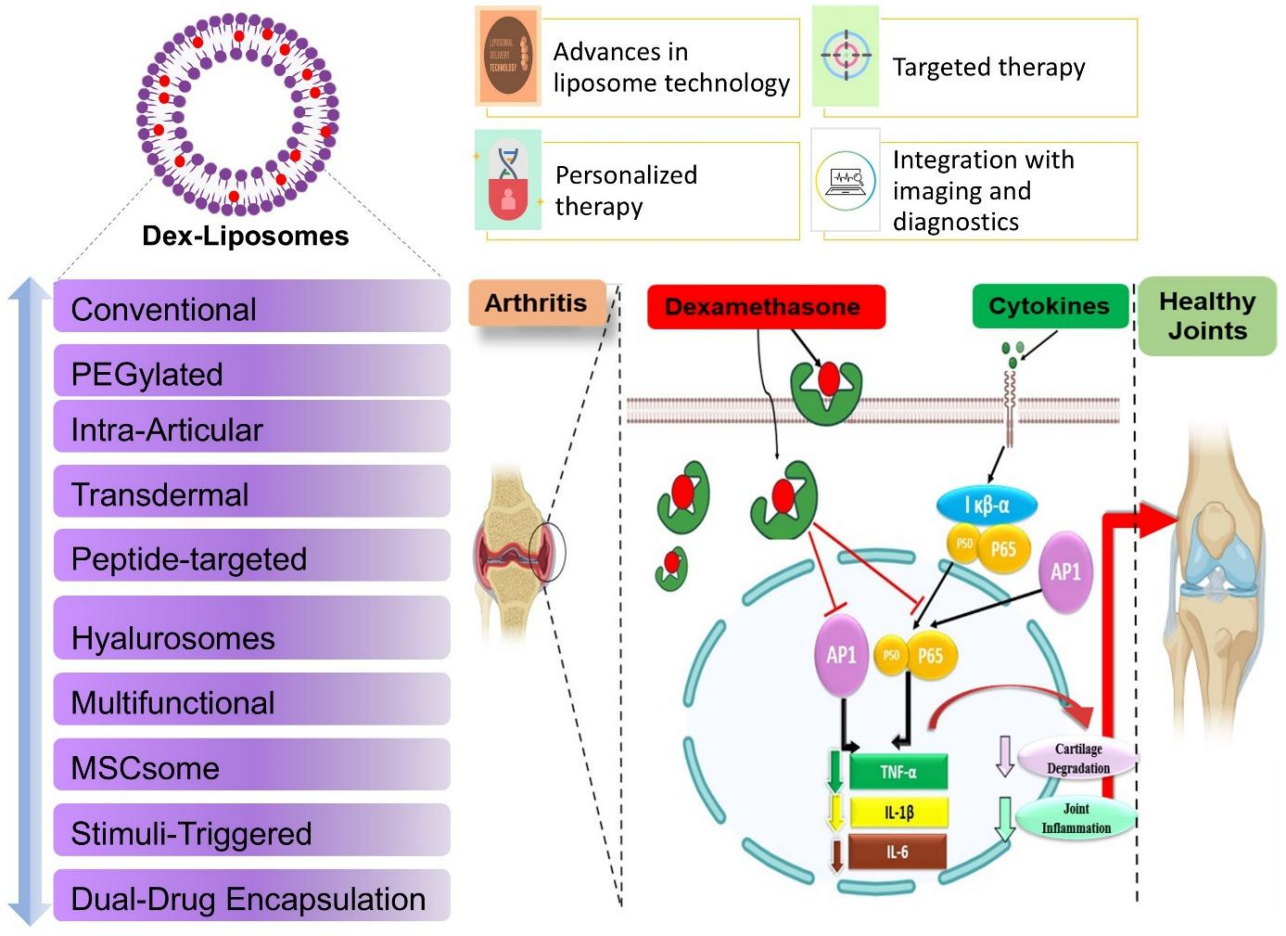
Graphical abstract
